# On the Critical Heat Flux Assessment of Micro- and Nanoscale Roughened Surfaces

**DOI:** 10.3390/nano12183256

**Published:** 2022-09-19

**Authors:** Uzair Sajjad, Imtiyaz Hussain, Waseem Raza, Muhammad Sultan, Ibrahim M. Alarifi, Chi-Chuan Wang

**Affiliations:** 1 Department of Energy and Refrigerating Air-Conditioning Engineering, National Taipei University of Technology, Taipei 10608, Taiwan; 2 Research Center of Energy Conversion for New Generation of Residential, Commercial and Industrial Sectors, National Taipei University of Technology, Taipei 10608, Taiwan; 3 Department of Mechanical Engineering, Jeju National University, Jeju 63243, Korea; 4 Department of Agricultural Engineering, Faculty of Agricultural Sciences & Technology, Bahauddin Zakariya University, Multan 60800, Pakistan; 5 Department of Mechanical and Industrial Engineering, College of Engineering, Majmaah University, Al-Majmaah, Riyadh 11952, Saudi Arabia; 6 Department of Mechanical Engineering, National Yang Ming Chiao Tung University, 1001 University Road, Hsinchu 300, Taiwan

**Keywords:** micro/nano roughness, pool boiling, critical heat flux, random forest, optimization, feature engineering

## Abstract

The boiling crisis or critical heat flux (CHF) is a very critical constraint for any heat-flux-controlled boiling system. The existing methods (physical models and empirical correlations) offer a specific interpretation of the boiling phenomenon, as many of these correlations are considerably influenced by operational variables and surface morphologies. A generalized correlation is virtually unavailable. In this study, more physical mechanisms are incorporated to assess CHF of surfaces with micro- and nano-scale roughness subject to a wide range of operating conditions and working fluids. The CHF data is also correlated by using the Pearson, Kendal, and Spearman correlations to evaluate the association of various surface morphological features and thermophysical properties of the working fluid. Feature engineering is performed to better correlate the inputs with the desired output parameter. The random forest optimization (RF) is used to provide the optimal hyper-parameters to the proposed interpretable correlation and experimental data. Unlike the existing methods, the proposed method is able to incorporate more physical mechanisms and relevant parametric influences, thereby offering a more generalized and accurate prediction of CHF (R^2^ = 0.971, mean squared error = 0.0541, and mean absolute error = 0.185).

## 1. Introduction

From previous understanding, CHF is considered the most critical constraint for heat-flux-controlled boiling systems. Pool boiling, as one of the simplest and most economical two-phase cooling techniques, is also well adopted in various applications, and it is applied for low and high temperature systems. In pool-boiling applications, such as immersion cooling for electronics cooling, the drastic rise in the surface temperature when exceeding CHF might have disastrous consequences [1]. Hence, the accurate estimation of CHF is critical. Investigators have been working for decades to develop prediction models and correlations for CHF, given its importance to cooling system designs [2].

As previously stated [2], pressure, surface orientation, and contact angle can all have a major impact on CHF. Most models and relationships account for pressure effects in thermal characteristics; properties like the vapor density and latent heat of vaporization are particularly pivotal. However, most original prediction methods do not account for the surface orientation and contact angle, and these predictions must therefore be adjusted to account for these effects. The fact that most predictive correlations were designed primarily for a horizontal, upward-facing surface orientation (0°) may limit the applicability of the prior correlations.

**Table 1:** Literature review on prediction methods of pool-boiling CHF.

Sr.	Ref.	CHF Prediction Method
1	[3]	Bubble interference model
2	[4,5,6]	Hydrodynamic instability model
3	[7,8]	Extended hydrodynamic theory
4	[9]	Macro layer dryout model
5	[10,11]	Hot/dry spot model
6	[12,13]	Interfacial lift-off model
7	[14]	Dimensional analysis
8	[15,16,17,18,19,20]	Correlations with effect of orientation at 1 atm.
9	[1,11,21,22]	Correlations with effects of orientation and contact angle at 1 atm.

While other approaches for accounting for additional surface orientations have been presented, the majority are completely empirical and based on data gathered solely at atmospheric pressure. Furthermore, most articles on pool-boiling CHF fail to discuss or even ignore the contact angle effects [23]. In particular, the appearance of modern manufacturing methods including additive, subtractive, compound, and other fabrication techniques have produced engineered surfaces with complex morphologies (dissimilar to the plain surfaces) [24,25,26,27]. This has further complicated the CHF prediction task, particularly for the conventional methods. 

Mudawar et al. [23] summarized the prediction methods of pool-boiling CHF (see Table 1). They recommended some of the methods with the lowest MAEs (mean absolute error) for different cases by evaluating various popular models and correlations used to predict pool-boiling CHF. The suggested CHF prediction methods for an upward-facing orientation are based on hydrodynamic theory [7,8] and an analytical model [12]. For atmospheric pressure and different orientations, the suggested models and correlations can be found in the literature [7,12,16]. For contact angles of 0° to 90°, Liao et al.’s correlation [22] and Kandlikar’s model [1] demonstrate a superior performance for different pressures and orientations. 

The existing CHF prediction correlations and methods are not universal; hence, they are valid only for a certain range. As a generalized model or correlation is virtually unavailable for the prediction of the CHF, the objective of the present study is to use machine learning and an artificial intelligence-based model to provide a generalized assessment of the CHF. Different models have proved their effectiveness, depending on the application [28,29,30], materials [31] and manufacturing techniques [32]. The appearance and adaptation of different fabrication methods and materials can be found in the literature [33,34,35]. For instance, data-driven models have been developed and tested to predict the heat transfer coefficient of sintered, coated and porous [36] as well as of roughened surfaces [37]. However, to the best of the authors’ knowledge, no attempt has been made to correlate the CHF data of roughened surfaces for a range of working fluids, substrates, morphologies, inclinations, and testing conditions.

Herein, a deep learning method is developed to assess the CHF of three classes of working fluids, including refrigerants, dielectric liquids, and water, subject to pool boiling with a variety of engineered surfaces and testing conditions. To ensure a highly accurate prediction of the CHF, the proposed method includes some of the most important pool-boiling parameters. In line with this, all the required information on the surface morphology, operating conditions, and important thermophysical properties of the considered working fluids is fed to the developed optimal neural network (DNN) to foresee the CHF. The optimal DNN framework is obtained by employing Bayesian surrogate models to finetune the hyper-parameters. The detailed methodology for selecting the important input parameters and hyper-parameters’ optimization and for developing the DNN model can be found in our prior works [38,39]. A wide range of pool-boiling CHF data is considered in this investigation. 

## 2. Methodology

**Figure 1:**
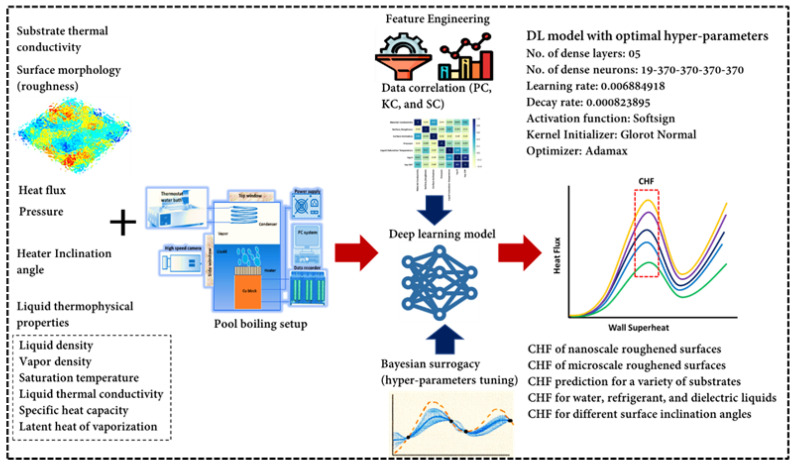
An overview of the research methodology.

**Figure 2:**
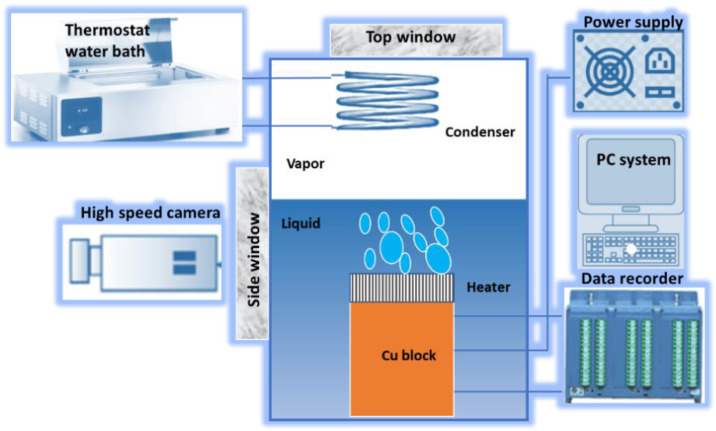
Schematic of the pool-boiling experimental setup.

**Figure 3:**
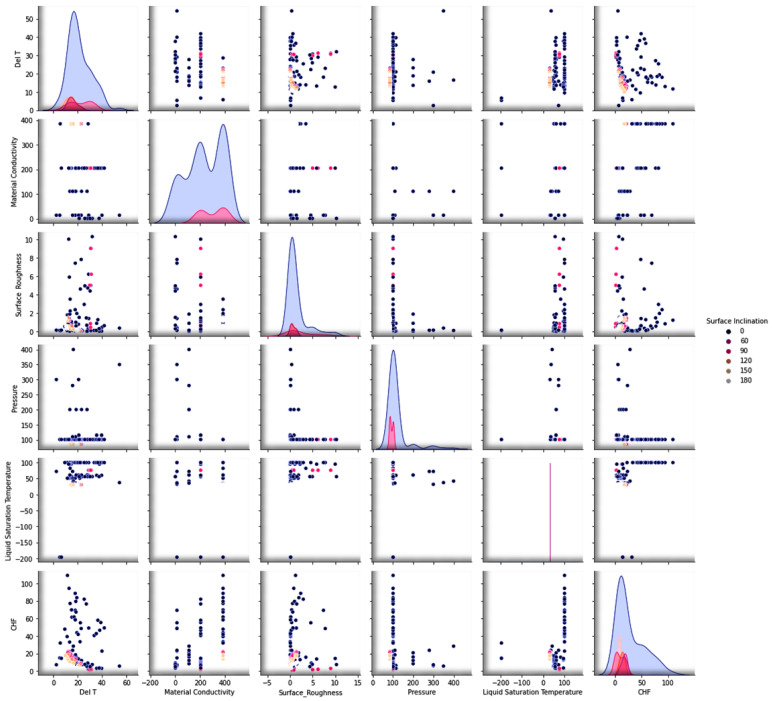
Visualizing the experimental CHF data in terms of the surface inclination, liquid saturation temperature, pressure, surface roughness, and material conductivity.

An overview of the detailed methodology including the experimental data collection, data visualization, data correlation, feature engineering, hyper-parameters’ tuning, and the development of the optimal deep learning model is given in Figure 1. The pool-boiling setup is illustrated in Figure 2. The experimental setup consists of a copper heating block, PEEK block (with a very low thermal conductivity), MX 100 data recorder system, thermostat reservoir, preheater, and high-speed camera for the boiling process visualization. A detailed description is available in our previous studies [40,41]. The pair plot in Figure 3 illustrates the scattering of the CHF and various inputs’ data with respect to each other. The data distribution is shown with respect to the heater surface inclination (horizontal facing upward, vertical, inclined, and horizontal facing downward), liquid saturation temperature, pressure, surface roughness, and material conductivity. Figure 3 represents the data distribution of the considered input and output parameters. Apparently, the data included in this investigation is scattered and dispersed, which shows that the task in hand (e.g., the CHF prediction) is quite complex and generalized. This is because a wide range of input and output variables are investigated in this study. Obviously, the applicability of most of the data is for a horizontal heater (facing upward) followed by a vertical and inclined one.

The Pearson correlation [42] is one of the most common statistical correlations. It determines the direction and degree of association between the considered variables. A non-parametric test called the Kendall rank correlation [43] assesses the degree of reliance between two variables. The Pearson product-moment correlation’s nonparametric counterpart is the Spearman’s rank-order correlation [44]. A non-parametric analysis called the Spearman rank correlation is employed to gauge how closely two variables are related. For the variables on the ordinal scale at least, this correlation provides a proper evaluation, as it does not assume the data distribution. 

**Figure 4:**
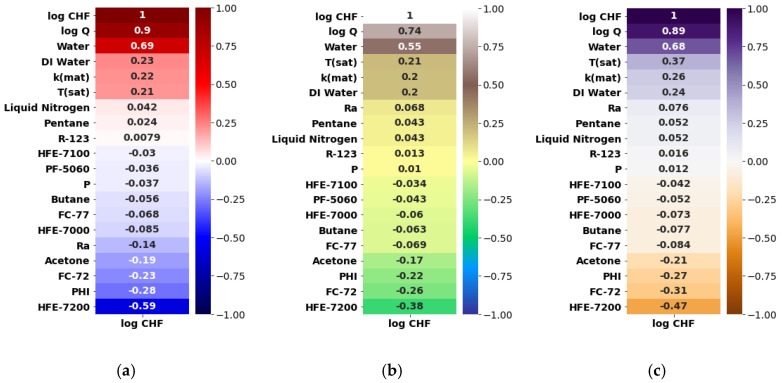
Correlating all CHF data with categorical variables: (**a**) Pearson correlation, (**b**) Kendal correlation, and (**c**) Spearman correlation.

**Figure 5:**
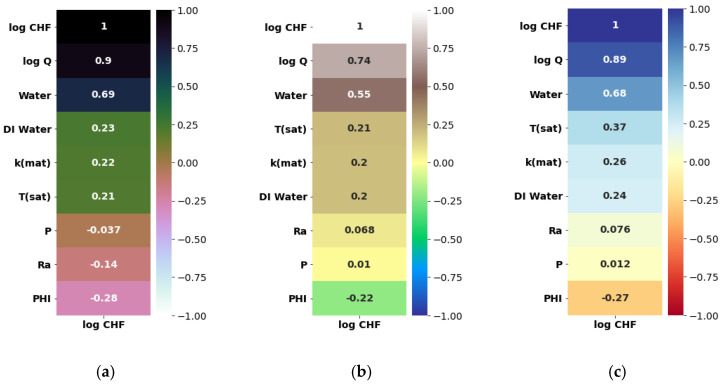
Correlating CHF data of water with categorical variables: (**a**) Pearson correlation, (**b**) Kendal correlation, and (**c**) Spearman correlation.

**Figure 6:**
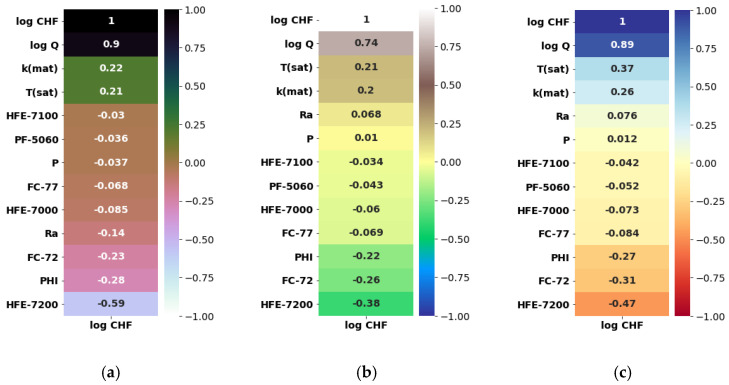
Correlating CHF data of dielectric liquids with categorical variables: (**a**) Pearson correlation, (**b**) Kendal correlation, and (**c**) Spearman correlation.

**Figure 7:**
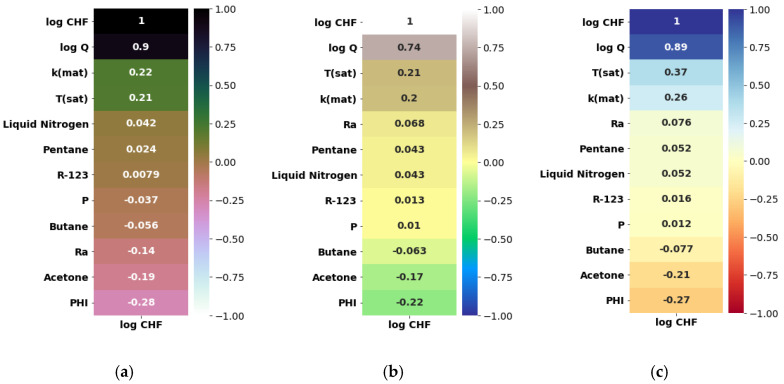
Correlating CHF data of refrigerants with categorical variables: (**a**) Pearson correlation, (**b**) Kendal correlation, and (**c**) Spearman correlation.

**Figure 8:**
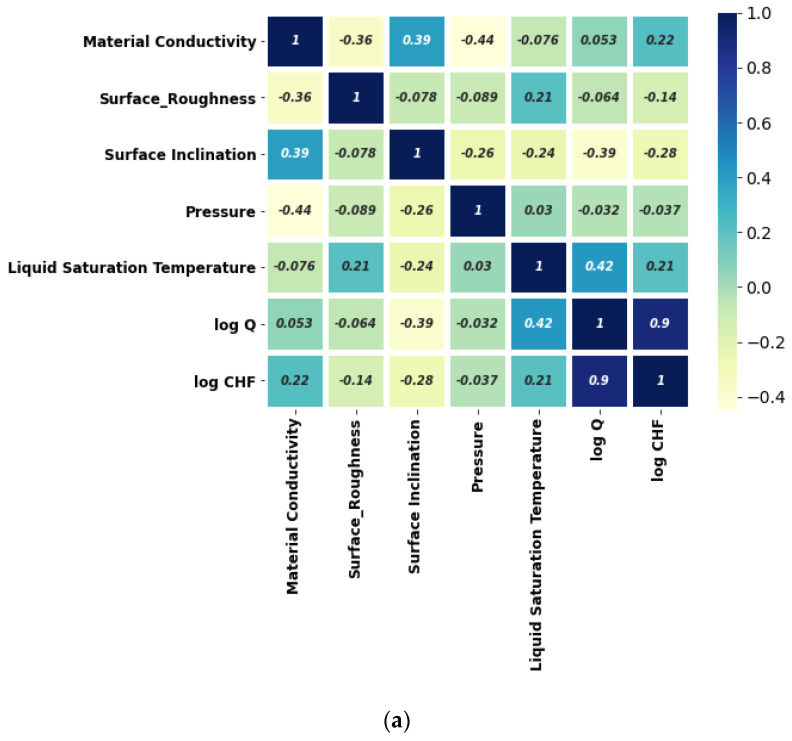

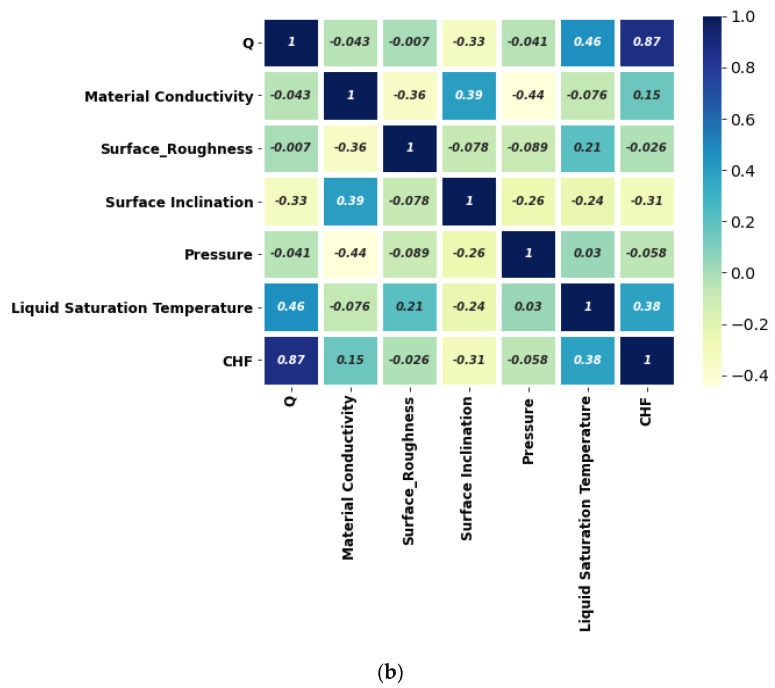
Normal heat map for CHF data correlation (**a**) without feature engineering and (**b**) with feature engineering.

**Figure 9:**
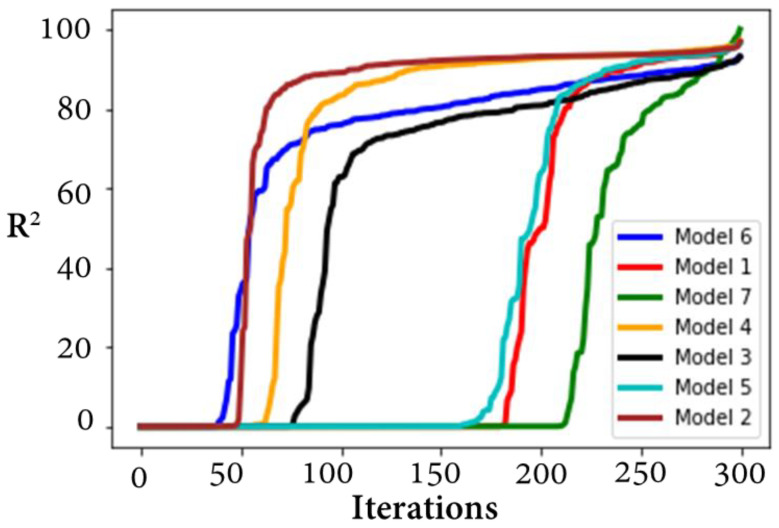
Illustration of the developed hyper-parameters tuning models.

Herein, the pool-boiling CHF data of the individual class of working fluids (such as water, refrigerant, and dielectric liquid) and their combination is assessed and correlated by using Pearson (PC), Kendal (KC), and Spearman (SC) correlations, as shown in Figure 4, Figure 5, Figure 6 and Figure 7. Different input variables including Q (logarithmic heat flux), T_sat_ (saturation temperature), K_mat_ (thermal conductivity of material), P (pressure), R_a_ (surface roughness), heater surface inclination (PHI), and some categorical variables are correlated with the CHF of the studied working fluids with these correlations. It can be observed that some of the inputs (Log Q, K_mat_, T_sat_, and R_a_) are positively related, while the others (P and PHI) have a negative impact on the CHF for the studied working fluids. All three correlations in Figure 4, Figure 5, Figure 6 and Figure 7 suggest that Q has a strong correlation (ranging between 0.74 to 0.9) with the CHF. For most of the inputs, these correlations show an almost similar degree and direction of the correlation (Figure 4, Figure 5, Figure 6 and Figure 7). For instance, the degree and magnitude of association of the material’s conductivity with the CHF shown by all three correlations is around +0.2. The degree of association of the liquid saturation temperature with the CHF shown by the Pearson and Kendal correlations is around +0.2, while the Spearman correlation shows this value to be +0.3. There might be some differences in the magnitude of the association of different inputs with the HCF; however, the direction of association shown by all three correlations is similar (see Figure 4, Figure 5, Figure 6 and Figure 7). This is valid for various classes of working fluids including water, refrigerants, and dielectric liquids. Furthermore, feature engineering proved to be quite helpful in providing a relatively better association between the inputs and the output, as highlighted in Figure 8. In Figure 8, two heat maps with and without feature engineering (Log transformation) depict slightly different correlations between the inputs and the CHF data. The shortcoming of the limited data was tackled by feature engineering. The log transformation was employed with different inputs and the output to better relate the data and improve the predictive accuracy of the model (see Figure 9 and Table 2).

From Figure 8, it can be noted that feature engineering (logarithmic transformation) is able to better relate the input variables of the surface, liquid, and testing conditions with the CHF. For instance, the degree of the association suggested by the heat map (with and without feature engineering) is different. A comparison shows that feature engineering is effective in increasing the accuracy of finding the correlation for the studied parameters (see Figure 9 and Table 2).

**Figure 10:**
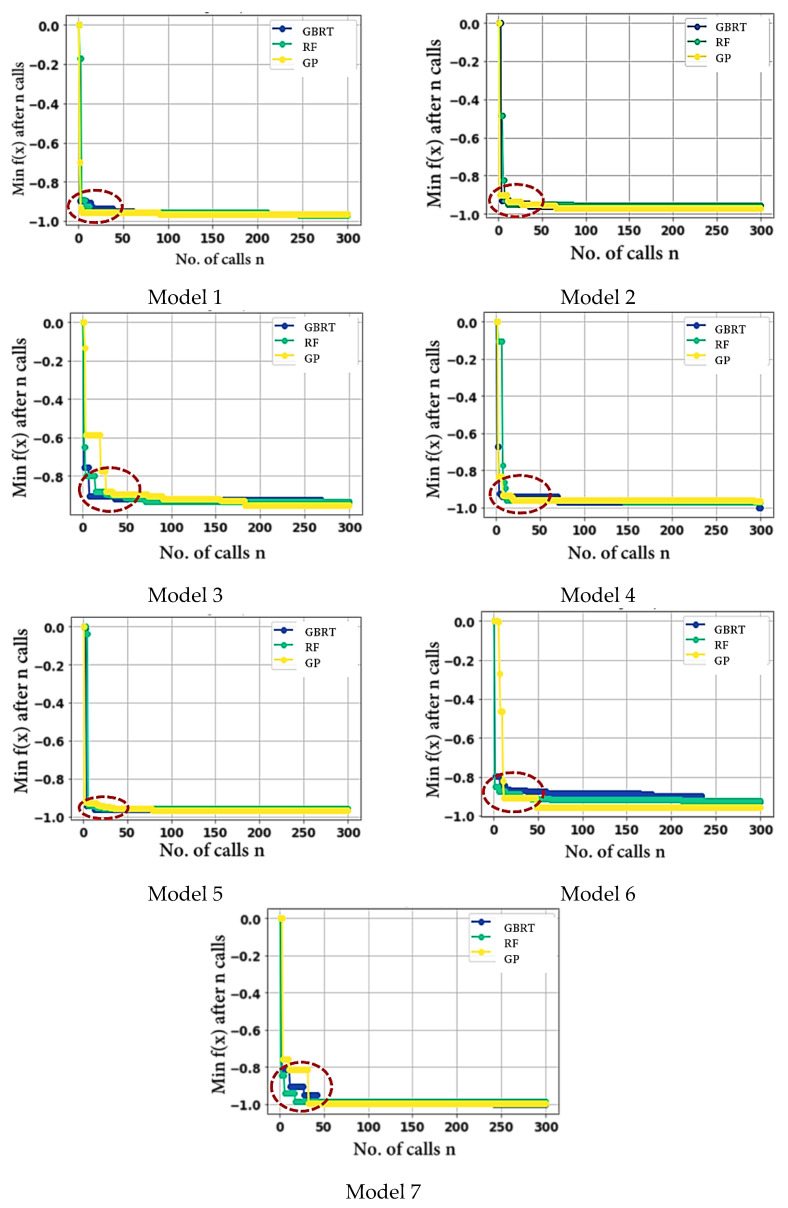
Convergence of the developed models 1–7.

**Figure 11:**
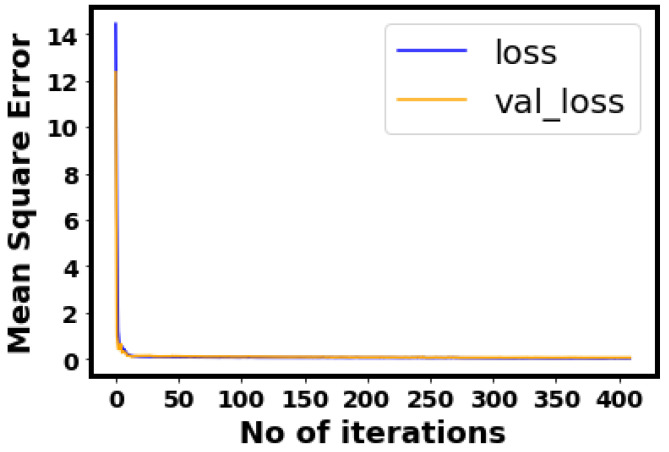
Training and validation losses’ minimization.

Herein, three hyper-parameters tuning methods, random forest (RF), gradient boosting regression trees (GBRT), and Gaussian process (GP), were evaluated to find the optimal tuning method. In this study, the CHF was correlated with heat flux (Q) and ΔT_sat_ separately to select the more influential input. Several methods were tried and evaluated in feature engineering. In the first case, Q is dropped, log is applied to CHF and Del T, and the resultant R^2^ are 96.95, 95.57, and 95.12 for RF, GP, and GBRT, respectively. In the second case, log is applied to all input features, resulting in an R^2^ of 95.03, 96.92, and 94.69 for RF, GP, and GBRT, respectively. The third case (Del T dropped) results in an R^2^ of 93.11, 91.11, and 92.13 for RF, GP, and GBRT, respectively. The fourth case (Del T is dropped, log is applied to CHF and Q) shows comparatively better results (R^2^ of 97.14, 97.05, and 97.06 for RF, GP, and GBRT, respectively). The procedure to calculate R^2^, RD, MSE, and MAE can be found in our prior study [45]. The rest of the cases are presented in Table 2. The convergence of the 07 models (all test cases) can be found in Figure 10. For all test cases of RF, GP, and GBRT, the convergence is achieved within the first few iterations. In test case 4, the model 4 shows relatively better results for random forest. This is also verified by the corresponding R^2^ values of the RF, GP, and GBRT. The optimal model, based on the hyper-parameters provided by model 4, has 05 dense layers (19-370-370-370-370). The learning and decay rates are 0.006884918 and 0.000823895, respectively. The employed activation function, kernel initializer, and optimizer are softsign, glorot normal, and adamax, respectively. The loss minimization (validation and training losses) is illustrated in Figure 11. It can be seen that within 20 iterations, MSE approaches zero for the training and validation losses. This shows the performance of the proposed method. The hyper-parameters tuning procedure is explained in a prior work [46].

## 3. Results and Discussion

The results and Discussion section consist of a comparison between the experimental and predicted data of the CHF with respect to the different input parameters (such as the material conductivity, surface roughness, surface inclination, operating pressure, liquid saturation temperature, and heat flux). Furthermore, this section includes the prediction performance of the developed model in terms of the heat map. Finally, the error density analysis is presented. 

**Figure 12:**
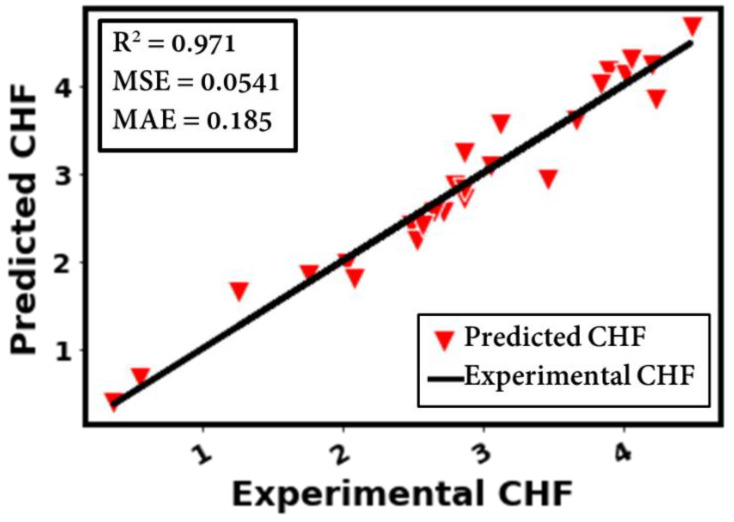
Prediction plot to assess the actual and anticipated data of the CHF.

**Figure 13:**
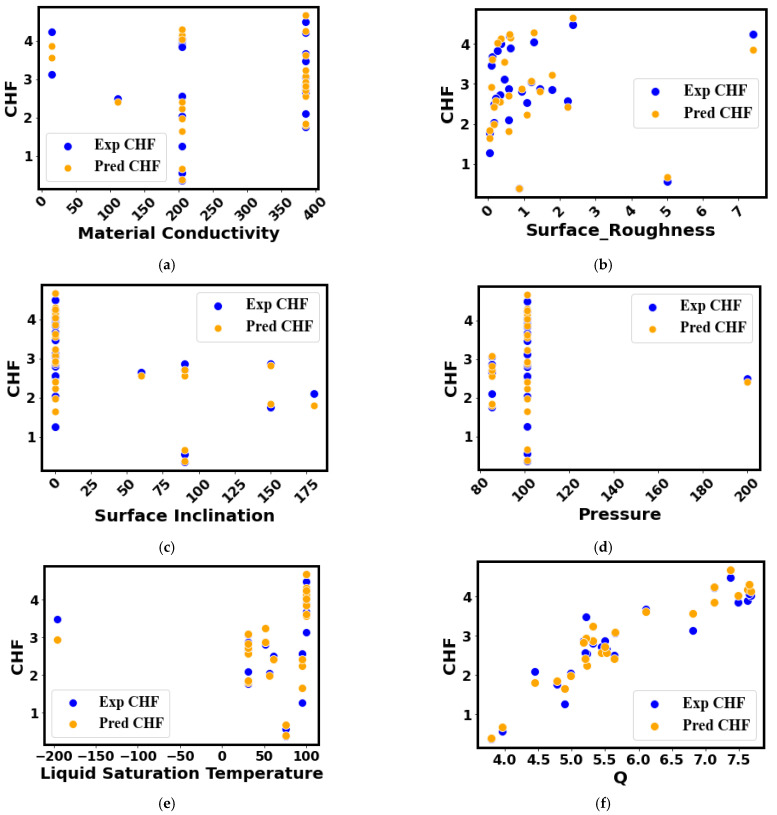
Prediction plots to assess the actual and anticipated data of the CHF with respect to the input parameters: (**a**) material conductivity, (**b**) surface roughness, (**c**) surface inclination, (**d**) pressure, (**e**) liquid saturation temperature, and (**f**) heat flux.

Figure 12 depicts the prediction performance of the developed method for the CHF of different surface morphologies, substrate materials, testing conditions, and working fluids. Additionally, the prediction results cover the different heater inclination angles during diverse pool-boiling conditions. An assessment of the actual and anticipated CHF data reveals a high accuracy (R^2^ = 0.971), relative deviation (RD) = 0.1%, mean squared error (MSE) = 0.0541, and mean absolute error (MAE) = 0.185 of the developed model, as illustrated in Figure 12. Clearly, the selected model accurately projects the CHF data throughout the investigated range. Such a high accuracy and extended application range imply the accurate correlation of the input parameters with the CHF data. Although the developed model is trained with limited data, the feature engineering and accurate data correlation along with the inclusion of an optimal set of hyper-parameters into the developed DL (deep learning) model make this method applicable for a wide range of pool-boiling conditions in terms of boiling liquids, heater surfaces and morphologies, and testing conditions. Figure 13 represents a comparison between the actual and anticipated data of the CHF with respect to the different input parameters: (a) material conductivity, (b) surface roughness, (c) surface inclination, (d) pressure, (e) liquid saturation temperature, and (f) heat flux. 

**Figure 14:**
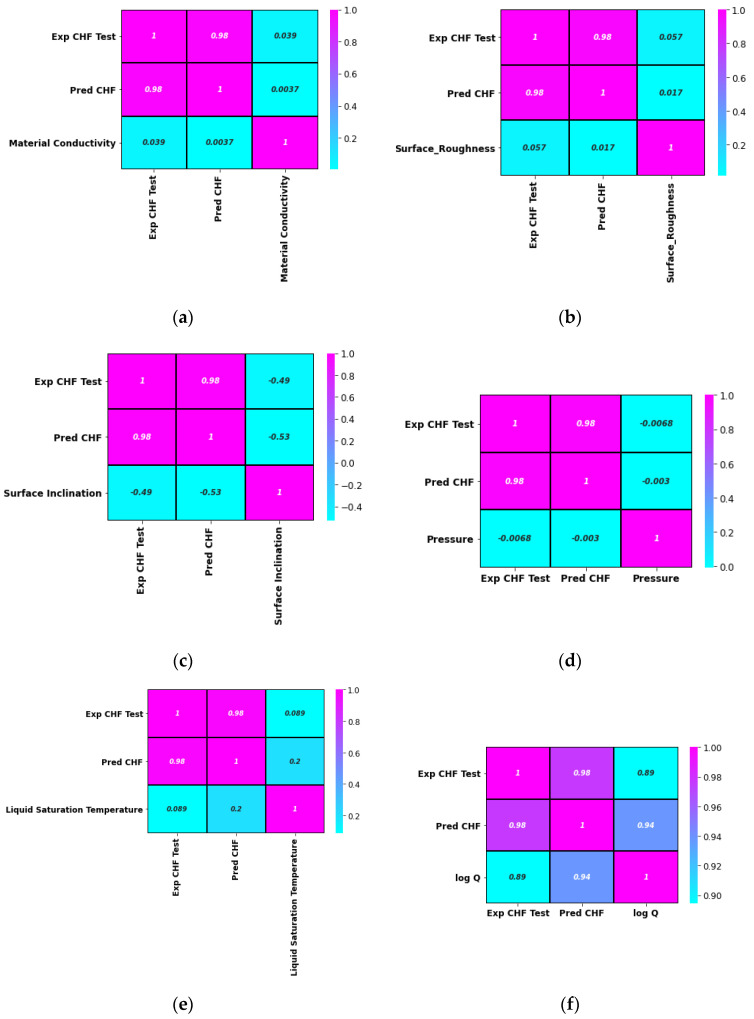
Heat map comparison plots to assess the actual and projected data of the CHF with respect to the input parameters: (**a**) material conductivity, (**b**) surface roughness, (**c**) surface inclination, (**d**) pressure, (**e**) liquid saturation temperature, and (**f**) heat flux.

In Figure 13a, it can be seen that the actual and projected points coincide with each other for different materials’ thermal conductivities ranging from 1 to 398 W/mK. In particular, the predictions for the CHF data of the highly thermally conductive surfaces (such as 200 and 398 W/mK) are highly accurate. Similar trends can be observed when viewing projections related to the other input parameters such as the pressure, surface inclination angle, etc. Once again, this can be attributed to the inclusion of the appropriate correlation between the inputs and the diverse CHF data. Figure 13b–f compare the predicted and experimental CHF data in terms of the surface roughness, surface inclination angle, pressure, liquid saturation temperature, and heat flux, respectively. The model’s predictions are compared with the experimental CHF values of different liquids and surfaces and are visualized in terms of heat map plots, as depicted in Figure 14. A close match between the experimental (1.0) and projected CHF (0.98) can be observed in Figure 14. 

**Figure 15:**
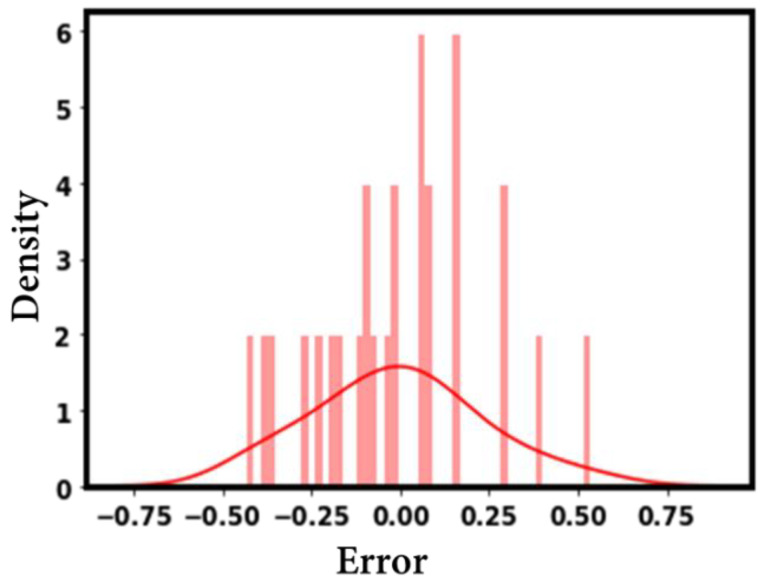
Error density representation.

The material thermal conductivity is positively correlated with the CHF, as shown in Figure 14a. Apart from the direction of association, Figure 14 reveals the degree of correlation between different input variables and the CHF. From Figure 14b, it is obvious that the surface roughness is positively correlated with the CHF. Furthermore, the roughness value has a strong impact on the CHF. Among the considered inputs, the pressure and surface inclination angle are negatively correlated with the CHF. This means that, for the investigated data range and conditions, higher values of the surface inclination angle and pressure will yield a lower CHF for the considered data on roughened surfaces (see Figure 14). The error density plot in Figure 15 shows that most of the CHF data are predicted with zero error, while only a few points are predicted with a slight error (error ranges between +0.75 and −0.75). Thus, Figure 15 demonstrates the superior prediction performance of the developed model.

One can understand that the proposed method can tackle the problem of predicting the CHF data of roughened surfaces (for a range of surface roughness values). The overall accuracy of the developed method in predicting the CHF is decent, with R^2^ = 0.971, RD = 0.1%, MSE = 0.0541, and MAE = 0.185.

## 4. Conclusions

An overview of the existing pool-boiling literature (empirical and semi-empirical models and correlations) clearly shows that virtually no generalized model or correlation is available to predict the CHF for different solid-liquid combinations. This study incorporates additional physical mechanisms to evaluate the critical heat flux (CHF) for surfaces with a micro- and nano-scale roughness due to a variety of operating circumstances and working fluids. The link between different surface morphological aspects and the thermophysical characteristics of the working fluid is further evaluated by using the Pearson, Kendal, and Spearman correlations on the CHF data. The best hyper-parameters for the suggested interpretable correlation and experimental data are provided using random forest optimization (RF). By including more physical mechanisms and pertinent parametric factors than in existing techniques, the suggested method provides a more precise and general CHF prediction. The major findings are summarized as follows:

1- With feature engineering (by using logarithmic transformation), different inputs (such as liquid thermophysical properties, surface morphology, and testing conditions) could be better correlated with the pool-boiling CHF. For instance, when log is applied to CHF and heat flux, a high accuracy (R^2^ = 0.971) is achieved.
2- Different correlations such as Pearson, Spearman, and Kendal correlations can be helpful in finding the degree and direction of association between the investigated features.
3- For hyper-parameters’ optimization, the RF model yields a relatively better accuracy compared to the GP and GBRT.
4- The optimal model (with 05 dense layers of 19-370-370-370-370 neurons, learning and decay rates of 0.006884918 and 0.000823895, and with the activation function, kernel initializer, and optimizer as softsign, glorot normal, and adamax, respectively) could achieve an accuracy of R^2^ = 0.971, RD = 0.1%, MSE = 0.0541, and MAE = 0.185.
5- The developed method is able to predict the CHF for a wide range of surface morphologies (nanoscale roughness in nm to microscale roughness in µm), substrate materials (copper, aluminum, stainless steel, etc.), and working fluids (refrigerants, dielectric liquids, and water). The investigated CHF ranges between 80 and 2079 kW/m^2^.
6- The CHF prediction model’s accuracy is valid for different heater inclination angles and operating pressures.

## Figures and Tables

**Table 2:** An overview of the feature engineering evaluation.

Sr.	Description	R^2^ of Bayesian Surrogate Models		
		RF	GP	GBRT
**1**	Q is dropped, log is applied to CHF and Del T	96.95	95.57	95.12
**2**	Log is applied to all input features	95.03	96.92	94.69
**3**	Del T is dropped	93.11	91.11	92.13
**4**	Del T is dropped, log is applied to CHF and Q	97.14	97.05	97.06
**5**	Del T is dropped, log is applied to CHF	96.22	95.44	96.40
**6**	Q is dropped	92.17	90.23	93.10
**7**	All features are included	98.5	100	99.8
